# Pedestrian-vehicle crashes: risk perception and responsibility attribution among children, adolescents and adults

**DOI:** 10.5249/jivr.v12i1.1243

**Published:** 2020-01

**Authors:** Sophie Yu, Yue Wu, Sylvie Mrug, Huarong Wang, Scarlett Ridley, Guoqing Hu, David C. Schwebel

**Affiliations:** ^*a*^ Birmingham, AL, USA.; ^*b*^ Department of Occupational and Environmental Health, Xiangya School of Public Health, Central South University, 110 Xiangya Road, Changsha 410078, China.; ^*c*^ Department of Psychology, University of Alabama at Birmingham. Campbell Hall 415, 1530 3rd Avenue South. Birmingham, AL 35294-1170.; ^*d*^ Department of Psychology, Institute of Nautical Medicine, Nantong University, 9 Seyuan Road, Nantong, Jiangsu Province 226019, China.; ^*e*^ Department of Epidemiology and Health Statistics, Xiangya School of Public Health, Central South University, 110 Xiangya Road, Changsha 410078, China.

**Keywords:** Unintentional pedestrian injuries, Risk perception, Responsibility attribution, Road safety, Child traffic safety

## Abstract

**Background:**

Child pedestrian injuries in China result from crashes not just with cars. We considered how Chinese youth and young adults perceive pedestrian risk from four vehicle types-bicycles, electric bicycles, cars, buses—evaluating perceptions for two factors that may influence pedestrian behavior and risk-taking, perception of road environment risk and responsibility to avoid crashes. Understanding how pedestrians perceive risk, and how these perceptions change as children grow older, could guide prevention efforts.

**Methods:**

383 children (grades 3-4, 5-6, 8) and university students completed self-report surveys. We analyzed overall responses, plus age/gender differences in risk perception and responsibility attribution, across vehicle types and number of vehicles approaching, using multivariate analysis of variance (MANOVA) and generalized estimating equation (GEE) models.

**Results:**

Overall, larger vehicles were perceived as riskier (p less than .001). Compared tochildren, university students perceived bicycles and electric bicycles as less risky (Mean=2.66 vs. 3.69, 3.34 vs. 3.62, respectively, p less than .05). Cars and buses were perceived as equally risky across age groups. Across all vehicle types and number of vehicles traversing the road, both children and young adults perceived more pedestrian responsibility to avoid collisions relative to drivers (p less than .001). Children attributed less personal responsibility to avoid pedestrian-vehicle crashes than university students (e.g., buses odds ratio (OR)=0.20, p less than .001; OR=0.26, p less than .01; OR=0.28, p less than .01 for third/fourth, fifth/sixth, eighth graders, respectively). University students and fifth/sixth graders also identified greater pedestrian responsibility to avoid collisions with multiple vs. one vehicle approaching (e.g., university students/cars OR=4.17, p less than .001).

**Conclusions:**

We discuss cognitive and perceptual development factors in childhood, adolescence, and young adulthood that may contribute to differences in risk perception and responsibility attribution among Chinese pedestrians and suggest future research should explore those processes and subsequently develop evidence-based interventions to reduce pedestrian injury risk.

## Introduction

Compared to high-income countries (8.2 mortalities per 100,000), China experiences high traffic injury rates (19.4 mortalities per 100,000).^1^ In fact, road traffic injuries represent the leading cause of injury death in China; as the most populous country in the world, China suffered nearly 262,000 road traffic deaths in 2017, accounting for about 21% of global road traffic deaths.^2^

Pedestrians are particularly vulnerable to road traffic crashes in China, accounting for over 40% of Chinese traffic fatalities.^3^ The pattern is particularly pronounced among children and young adults, who represent the focus of this study. Among those young people in China, pedestrian injuries account for over half of road traffic fatalities.^3^ While many people assume pedestrian injuries are caused only by crashes between pedestrians and large vehicles, almost half of nonfatal pedestrian injuries in urban Guangdong Province originated not from collisions with cars, but from other moving vehicles, including bicycles (26.3%), motorcycles/electric bicycles (19.0%), and tricycles (13.8%).^4^

How pedestrians perceive and respond to various types of moving vehicles is poorly understood but merits exploration because underestimation of risk may lead to riskier behaviors.^5^ Thus, if pedestrians judge bicycles, motorcycles, or tricycles as less risky, they may behave differently near them. This study examines, therefore, how Chinese children and young adults perceive pedestrian risk from various types of vehicles. In particular, we evaluated perceptions of two factors that may influence pedestrian behavior around vehicles: risk perception in the road environment and perception about responsibility to avoid crashes. We considered both general risk and developmental change in perception of risk across children and young adults.

Risk perception, conceptualized as the perceived vulnerability to a threat, drives a wide range of health promotion behaviors,^6-7^ including road traffic behaviors,^5^ and develops as children grow into adolescence and adulthood.^8^ Risk perception influences pedestrian safety because pedestrians who perceive risk may be more attentive and reactive to potential road hazards, whereas those who do not perceive risk may engage with traffic conditions beyond what they can manage safely.^9^

Perception about a pedestrian’s responsibility to avoid crashes may also influence pedestrian behavior and risk. Responsibility and risk judgments, which also evolve as individuals grow older and gain greater cognitive sophistication,^8^ motivate a wide range of behavioral reactions. For example, charity giving is more likely among adults if recipients are perceived as not responsible for their suffering, such as for hurricane victims rather than people who abuse drugs.^10-11^ In the case of pedestrian behavior, pedestrians may therefore take more risk if they feel they are not responsible to keep themselves safe and may behave more cautiously if they judge themselves responsible to maintain their own safety. Legally, Chinese policy requires drivers to yield to pedestrians in marked crosswalks, although such yielding is rarely practiced^12^ and the law is poorly enforced.^13^

Epidemiological data offer evidence of age-related differences in pedestrian mortality and morbidity,^3,14^ with child-related differences typically attributed to a range of cognitive, perceptual, and exposure-related factors^15-18^ that may not be fully developed until about age 14.^19-20^ Children likely overestimate their ability to keep safe in traffic given overestimation in other domains of functioning, including road-crossing skill and perception of speeds and distances of oncoming traffic,^15,21-22^ but children’s perception of risk in pedestrian environments has not been explored carefully in previous research, nor has the process of how child and adolescent development may influence children’s perception of risk in pedestrian environments. We addressed these research questions in the present study, hypothesizing that young adults and older children, who have more advanced cognitive processing skills and also more experience in traffic, would correctly recognize larger vehicles as more dangerous than smaller vehicles given the large vehicles’ greater mass, lack of maneuverability and need for greater braking distances. This hypothesis is grounded in classic Piagetian child development theory and Gibsonian ecological theory, as well as more contemporary writings on how children’s cognitive-perceptual-motor development influences their engagement in traffic^23^ and empirical work in related areas (e.g., how children and adults judge natural vs. anomalous collisions between objects;^24^ velocities of movement in toy vehicles; ^25^ and safety of crossing in dynamic traffic environments. ^9^ As a secondary topic of interest, we also hypothesized older children and adults might judge greater risk when vehicles move more quickly.

Moreover, we hypothesized that young adults and older children would attribute more responsibility for crash avoidance to themselves than would younger children. In particular, those children who do not have skills (or experience) to consider the driver’s perspective, including the capacity of a driver to manipulate or stop the vehicle, might inaccurately presume drivers can easily avert pedestrian crashes.^26^ We also hypothesized that pedestrians would perceive more responsibility to avoid collisions with many vehicles approaching toward them than when there was just one vehicle approaching, across all vehicle types, as it is easier to negotiate a road-crossing safely with a single oncoming vehicle than with densely approaching traffic. Last, we considered pedestrian gender. We hypothesized females would assign greater responsibility for pedestrian-vehicle crashes to pedestrians than males because previous research suggests that females are more likely to blame themselves for injuries than males,^27^ and that men may have more optimistic judgments of risk, including in traffic.^28-29^

In summary, we evaluated two perceptual factors that have not been studied carefully in previous research but may influence pedestrian behavior and therefore pedestrian injuries and deaths: perception about risk in the road environment, especially with respect to different types of vehicles, and perception about motorist vs. pedestrian responsibility to avoid crashes. We considered both perceptions overall among Chinese children and young adults as well as considering age- and gender-specific differences in those perceptions. We studied three age groups of children, those who are still learning to be safe pedestrians (grades 3-4), those who are beginning to master pedestrian safety (grades 5-6), and those who should have nearly mastered the capacity to engage safely in traffic (grade 8), plus a group of young adults presumed to be fully safe pedestrians. We posited three primary hypotheses: 1) young adults and older children will rate larger road vehicles as more risky than smaller vehicles but younger children will not; 2) perception of pedestrian responsibility to avoid collision with road vehicles will increase with age; and 3) across all age groups, greater pedestrian responsibility to avoid pedestrian-vehicle crashes will be perceived when multiple vehicles are approaching compared to when just one vehicle (of the same type) is approaching. 

## Methods 

*Participants.* A total of 395 participants were recruited from a convenient public primary school (grades three through six), public middle school (eighth grade) and university (university students) in Changsha, China. We selected these child age groups to gather data from the developmental stages when cognitive skills relevant to safe pedestrian behavior are believed to develop (roughly ages 8-14).^19,30^ The group of university students was recruited as a comparison group of adults, who are presumably competent pedestrians with fully-developed cognitive capacity to cross streets safely.

Data from two participants were dropped from analysis due to incomplete demographic information and data from 10 participants were excluded due to invalid completion of questionnaires (e.g., answering with the same response for all items). The final sample consisted of 383 participants. The sample was 94% Han Chinese ethnicity and 55% male, and included 97 third- and fourth-grade children (age Mean (M)=9.18 years; Standard Deviation (SD)=0.60), 111 fifth- and sixth-grade children (M=11.10 years; SD=0.79), 107 eighth-grade children (M=13.60 years; SD=0.43), and 68 university students (M=19.39 years; SD=0.55). The study protocol was approved by the Institutional Review Boards at both University of Alabama at Birmingham, United States, and Central South University, China. All participants provided informed consent.

*Measures.* All participants completed a self-report survey composed of 3 parts: (a) demographics, (b) risk perception, and (c) perception of responsibility in pedestrian-vehicle crashes. Surveys were completed in group format in classroom settings. 

Demographic information included gender, age, grade (omitted for university students), and ethnicity.

Risk Perception was measured through questions that asked “How risky is it to step in front of X vehicle?” with different items for bicycles, electric bicycles, cars and buses. For the last three vehicle types, which are capable of traveling fast, participants rated riskiness for the specific vehicle traveling at fairly slow (20 kilometers per hour [kph]) or moderately fast (30 kph) speeds. Responses were provided using a 5-point scale ranging from 1 (not risky at all) to 5 (extremely risky). Contrary to our secondary hypothesis, there were no statistically significant differences between perceptions of risk at 20 kph versus 30 kph, so the responses for those items were averaged to form a composite variable for each vehicle type. 

Perception of pedestrian responsibility was assessed via photographs of scenes on Chinese roadways that showed pedestrian crossings. Each picture differed across two variables: the type of vehicle traveling on the road (bicycle, electric bicycle, car, or bus) and the number of vehicles traveling on the road (one or many). Eight photographs in total were used therefore (4 types of vehicles × 2 numbers of vehicles), and participants responded to the question, “Who would be more responsible for avoiding collision?” with two options as answers: pedestrian or riders/drivers, for each photograph. Potential covariates such as background scenery and distances of the vehicle to the crosswalk were similar across all photographs.

Questionnaire items were developed by the research team and reviewed by multiple content experts for face validity prior to implementation. Translation from English to Chinese was conducted using standard translation/back-translation processes and all surveys were completed in Chinese.

*Data Analysis.* Data analysis proceed in three steps. First, descriptive statistics were generated overall as well as across grades and gender to assess perception of risk and pedestrian vs. driver responsibility to avoid traffic crashes for the four types of vehicles. Perceptions of risk across the four vehicle types were compared with paired samples t-tests. Attributions of responsibility (pedestrian vs. driver) for all vehicle types and number of vehicles were tested with binomial tests to indicate whether they differed from equal responsibility (50% each pedestrian and driver). 

Second, we considered risk perceptions. To evaluate age and gender differences in risk perceptions, a two-way multivariate analysis of variance (MANOVA) was computed with gender and grade as independent variables and the four perceived risk variables (bicycles, electric bicycles, cars, and buses) as dependent variables. Both main effects and interactions of grade and gender were tested. Significant effects were followed up with univariate analyses of variance (ANOVAs), with post-hoc comparisons across grades using Tukey HSD tests. 

The last step of data analysis considered responsibility attributions. To test grade and gender differences in the categorical indices of responsibility attributions (pedestrian vs. rider/driver), generalized estimating equation (GEE) models with binomial distribution were utilized to account for the repeated nature of the data. The GEE models were conducted separately for each vehicle type, with pedestrian responsibility as the dependent variable, age group and gender as between-subject predictors, and number of vehicles and the interaction of grade and number of vehicles as within-subject predictors. The interactions of grade with number of vehicles were interpreted using simple slopes (i.e., the main effect of number of vehicles for the reference age group).^31^ The interaction between age and gender was also explored, but it was not significant in any of the models and thus was removed from the final analyses. Analyses were conducted using SPSS Version 25 and SAS 9.4.

## Results

*Preliminary Analyses.* Descriptive statistics are presented in Table 1. Across the full sample, perception of pedestrian risk increased with increasing vehicular size. Based on paired samples t-tests, all pairwise comparisons across the four vehicle types were significant (t(382)≥6.19, p<.001). Participants gave bicycles the lowest average score of 3.32 (SD=.99) for riskiness, followed by 3.62 (SD=.74) for electric bicycles, 3.96 (SD=.81) for cars, and 4.22 (SD=.89) for buses. For responsibility attributions, participants were more likely to perceive pedestrians as responsible to avoid collisions instead of drivers across all vehicle types and number of vehicles approaching on the roadway (ranging from 59% pedestrian responsibility with one car approaching to 80% pedestrian responsibility with many buses approaching). Binomial tests suggested all of the proportions differed from 50% (all p<.001).

**Table 1:** Descriptive and Inferential Statistics—Risk Perception and Responsibility Attribution of Chinese Pedestrians.

Mean (SD) unless other-wise noted	Total	Third/Fourth Grades	Fifth/Sixth Grades	Eighth Grade	University	Males	Females
Participants (N)	383	97	111	107	68	211	172
Age	12.85 (3.55)	9.18 (0.60)	11.10 (0.79)	13.60 (0.43)	19.39 (0.55)	12.74 (3.46)	12.97 (3.67)
Risk Perception Rating ^1^							
Bicycle	3.32 (0.99)	3.69 (0.10)^a^	3.42 (0.09)^ab^	3.33 (0.09)^b^	2.66 (0.11)^c^	3.29 (1.06)	3.36 (0.90)
Electric Bicycle	3.62 (0.74)	3.62 (0.07)^a^	3.73 (0.07)^a^	3.67 (0.07)^a^	3.34 (0.09)^b^	3.65 (0.81)	3.59 (0.64)
Car	3.96 (0.81)	4.01 (0.08)^a^	3.96 (0.08)^a^	3.99 (0.08)^a^	3.79 (0.10)^a^	4.00 (0.83)	3.91 (0.78)
Bus	4.22 (0.89)	4.27 (0.09)^a^	4.12 (0.08)^a^	4.36 (0.09)^a^	4.05 (0.11)^a^	4.22 (0.92)	4.22 (0.85)
Pedestrian vs. Driver Responsibility Rating ^2^							
Bicycle - One	0.74 (0.44)	0.74 (0.44)	0.62 (0.49)	0.79 (0.41)	0.84 (0.37)	0.72 (0.45)	0.76 (0.43)
Bicycle - Many	0.75 (0.43)	0.72 (0.45)	0.80 (0.40)	0.72 (0.45)	0.84 (0.37)	0.79 (0.41)	0.74 (0.44)
Electric Bicycle - One	0.66 (0.47)	0.61 (0.49)	0.54 (0.50)	0.77 (0.42)	0.72 (0.45)	0.63 (0.48)	0.69 (0.47)
Electric Bicycle - Many	0.77 (0.42)	0.71 (0.46)	0.80 (0.40)	0.71 (0.46)	0.90 (0.31)	0.75 (0.43)	0.79 (0.41)
Car - One	0.59 (0.49)	0.60 (0.49)	0.50 (0.50)	0.61 (0.49)	0.68 (0.47)	0.57 (0.50)	0.61 (0.49)
Car - Many	0.74 (0.44)	0.61 (0.49)	0.80 (0.40)	0.67 (0.47)	0.90 (0.31)	0.72 (0.45)	0.75 (0.43)
Bus - One	0.75 (0.43)	0.66 (0.48)	0.73 (0.44)	0.74 (0.44)	0.91 (0.29)	0.71 (0.45)	0.80 (0.40)
Bus - Many	0.80 (0.40)	0.65 (0.48)	0.83 (0.38)	0.79 (0.41)	0.99 (0.12)	0.79 (0.41)	0.82 (0.38)

1 Risk perception was coded on 5-point scale; 1 signified not risky at all and 5 signified extremely risky. 2 Pedestrian responsibility to avoid collision was coded as 1; driver coded as 0. abc Age cohort groups with different superscripts differed from each other on risk perceptions of vehicle in that row. SD=standard deviation. SD=standard deviation.

*Risk perceptions.* Next, the effects of age and gender on risk perceptions were examined (see Table 1). The MANOVA produced a statistically significant interaction effect between age group and gender on the combined dependent variables, F(12, 985)=1.80, p=.043, Wilks' Λ=.94. Statistically significant interaction effects between age and gender were observed for risk perception of electric bicycles (F(3, 375)=4.09, p=.007), cars (F(3, 375)=4.47, p=.004), and buses (F(3, 375)=3.40, p=.018), but not bicycles (F(3, 375)=1.30, p=.275). Follow up tests indicated that males and females did not differ in their risk perceptions in third/fourth and eighth grade (all p>.05). In fifth/sixth grade, males reported greater risk for cars than females (p=.036) but did not differ in risk perceptions of electric bikes and buses (p>.09), and among university students females reported greater risk perceptions than males for all three vehicle types (all p <.007).

Additionally, there were statistically significant differences among age groups in the risk perceptions of bicycles (F(3, 375)=16.87, p<.0005) and electric bicycles (F(3, 375)=4.56, p=.004), but not cars (F(3, 375)=1.23, p=.300) or buses (F(3, 375)=2.32, p=.075). Table 1 indicates pairwise differences in the risk perception scores for the four age cohorts. While no significant differences emerged among age groups for cars and buses, both adolescents and young adults judged the smaller bicycles and just young adults judged the electric bicycles as less risky compared to the younger children. There was no significant main effect of gender in the MANOVA, F(4, 372)=16.87, p=.540, Wilks' Λ=.992.

*Responsibility attributions.* GEE models examining age differences for pedestrian responsibility to avoid collision separately for each type of vehicle also showed no gender differences (all p>.085) and no interactions of gender with age, indicating that males and females across all ages made similar attributions of pedestrian responsibility. However, a number of age differences and interactions between age and the number of vehicles emerged for each vehicle type (See Table 2). For bicycles, eighth graders and university students were more likely to attribute responsibility to pedestrians than fifth/sixth graders (OR=1.95 and 2.92 respectively). Pedestrian responsibility was increased by the presence of multiple bicycles (vs. one bicycle) for fifth/sixth graders only (OR=1.90). For electric bicycles, greater pedestrian responsibility was also reported by older individuals: eighth graders reported more pedestrian responsibility than both third/fourth graders and fifth/sixth graders (OR=2.09 and 2.70), and university students more than fifth/sixth graders (OR=2.08). Multiple vs. one electric bicycle increased pedestrian responsibility perceptions in fifth/sixth graders (OR=3.28) and university students (OR=3.38), but not in third/fourth or eighth graders. For cars, university students were more likely to endorse pedestrian responsibility than fifth/sixth graders (OR=2.02). The presence of multiple vs. one car increased pedestrian responsibility perceptions for fifth/sixth graders (OR=4.04) and university students (OR=4.17), but not third/fourth or eighth graders. Finally, for buses, university students were more likely to attribute responsibility to pedestrians than all three youth groups (OR=4.90 vs. third/fourth grades; OR=3.78 vs. fifth/sixth grades; and OR=3.60 vs. eighth grade); the other three groups did not differ from one another. The presence of multiple buses vs. one bus increased pedestrian responsibility attributions for fifth/sixth graders (OR=1.88) and university students (OR=6.51), but not for third/fourth and eighth graders.

**Table 2:** GEE Analyses of Pedestrian Responsibility Across Vehicle Types.

	Bicycle		Electric Bicycle		Car		Bus	
	b (SE)	OR	b (SE)	OR	b (SE)	OR	b (SE)	OR
3rd &4th Grades	-0.65 (0.40)	0.52	-0.47 (0.34)	0.62	-0.29 (0.34)	0.75	-1.59 (0.48)***	0.20
5th &6th Grades	-1.07 (0.38)**	0.34	-0.73 (0.33)*	0.48	-0.70 (0.32)*	0.49	-1.33 (0.48)**	0.26
8th Grade	-0.40 (0.40)	0.67	0.26 (0.36)	1.30	-0.30 (0.33)	0.74	-1.28 (0.48)**	0.28
University	ref		ref		ref		ref	
Female	-0.01 (0.17)	0.99	0.19 (0.19)	1.21	0.16 (0.19)	1.18	0.38 (0.22)	1.46
Many	0.00 (0.49)	1.00	1.22 (0.40)**	3.38	1.43 (0.43)***	4.17	1.87 (0.93)*	6.51
3rd &4th Grades*Many	-0.06 (0.58)	0.95	-0.80 (0.48)	0.45	-1.36 (0.50)**	0.26	-1.93 (0.95)*	0.15
5th &6th Grades*Many	0.64 (0.58)	1.90	-0.03 (0.49)	0.97	-0.03 (0.50)	0.97	-1.24 (0.96)	0.29
8th Grade*Many	-0.39 (0.56)	0.68	-1.57 (0.48)***	0.21	-1.13 (0.48)*	0.32	-1.61 (0.96)	0.20
3rd&4th Grades	ref		ref		ref		ref	
5th& 6th Grades	-0.42 (0.30)**	0.66	-0.26 (0.29)	0.77	-0.42 (0.29)	0.66	0.26 (0.31)	1.30
8th Grade	0.25 (0.33)	1.28	0.74 (0.34)*	2.09	-0.02 (0.29)	0.98	0.31 (0.31)	1.36
University	0.65 (0.40)	1.92	0.47 (0.34)	1.61	0.29 (0.34)	1.33	1.59 (0.48)***	4.90
Many	-0.06 (0.32)	0.95	0.42 (0.27)	1.52	0.07 (0.26)	1.07	-0.06 (0.23)	0.95
3rd &4th Grades*Many	0.70 (0.44)	2.00	0.77 (0.39)*	2.16	1.33 (0.36)***	3.78	0.69 (0.35)*	1.99
5th &6th Grades*Many	-0.33 (0.42)	0.72	-0.77 (0.37)*	0.46	0.23 (0.33)	1.26	0.32 (0.34)	1.38
8th Grade*Many	0.06 (0.58)	1.06	0.80 (0.48)	2.22	1.36 (0.50)**	3.91	1.93 (0.95)*	6.88
3rd &4th Grades	0.42 (0.30)	1.52	0.26 (0.29)	1.29	0.42 (0.29)	1.52	-0.26 (0.38)	0.77
5th &6th Grades	ref		ref		ref		ref	
8th Grade	0.67 (0.30)*	1.95	0.99 (0.30)**	2.70	0.40 (0.28)	1.49	0.04 (0.31)	1.05
University	1.07 (0.38)**	2.92	0.73 (0.33)*	2.08	0.70 (0.32)*	2.02	1.33 (0.48)**	3.78
Many	0.64 (0.30)*	1.90	1.19 (0.28)***	3.28	1.40 (0.24)***	4.04	0.63 (0.26)*	1.88
3rd &4th Grades*Many	-0.70 (0.44)	0.50	-0.77 (0.39)*	0.46	-1.33 (0.36)***	0.26	-0.69 (0.35)*	0.50
5th &6th Grades*Many	-1.03 (0.40)*	0.36	-1.54 (0.38)***	0.21	-1.10 (0.32)***	0.33	-0.37 (0.36)	0.69
8th Grade*Many	-0.64 (0.57)	0.53	0.03 (0.49)	1.03	0.03 (0.50)	1.03	1.24 (0.96)	3.46
3rd &4th Grades	-0.25 (0.33)	0.78	-0.74 (0.31)*	0.48	0.02 (0.29)	1.02	-0.31 (0.31)	0.73
5th & 6th Grades	-0.67 (0.30)*	0.51	-0.99 (0.30)**	0.37	-0.40 (0.28)	0.67	-0.04 (0.31)	0.96
8th Grade	ref		ref		ref		ref	
University	0.40 (0.40)	1.49	-0.26 (0.36)	0.77	0.30 (0.33)	1.35	1.28 (0.48)**	3.60
Many	-0.39 (0.27)	0.68	-0.35 (0.26)	0.70	0.29 (0.20)	1.34	0.26 (0.25)	1.30
3rd &4th Grades*Many	0.33 (0.42)	1.39	0.77 (0.37)*	2.16	-0.23 (0.33)	0.79	-0.32 (0.34)	0.73
5th &6th Grades*Many	1.03 (0.40)*	2.80	1.54 (0.38)***	4.66	1.10 (0.32)***	3.00	0.37 (0.36)	1.45
8th Grade*Many	0.39 (0.56)	1.48	1.57 (0.48)***	4.81	1.13 (0.48)*	3.10	1.61 (0.96)	5.00

*p<05; **p <01; ***p <001. SE=standard error; OR=odds ratio; ref=reference. Significant results are bolded. Gender and grade are between-subject pre-dictors. Vehicle type, number of vehicles (many vs. one), and interactions between vehicle type and number are within-subject predictors. Note that each segment of the table presents analyses with different grades used as reference groups. All pairwise comparisons and simple slopes are reported for analyses of interaction effects.

## Discussion

Our analysis yielded numerous noteworthy results. First, study participants in all age groups perceived that the smaller vehicles we considered, bicycles and electric bicycles, were less risky vehicles on the roads compared to cars and buses, although young adults tended to perceive all vehicles as less risky than children. Second, across all vehicle types and number of vehicles traveling on the road, both children and young adults perceived more responsibility on the part of pedestrians to avoid collisions relative to drivers. Children tended to ascribe less responsibility to pedestrians to avoid crashes than young adults did. Last, we found that university students and fifth/sixth graders identified greater pedestrian responsibility to avoid collisions when there were multiple vehicles approaching rather than just one vehicle for most vehicle types. We discuss each of these findings below.

We hypothesized that young adults and older children would judge larger road vehicles as riskier than smaller vehicles, whereas younger children might rate all vehicles similarly. Our results suggest this hypothesis was not supported. In fact, all participants identified higher risk from larger vehicles compared to smaller vehicles, although adolescents and young adults judged the smaller bicycles and young adults judged the electric bicycles as less risky compared to the younger children. Our results may reflect the possibility that even the youngest children in the sample – those in third and fourth grade – were able to perceive the risk of experiencing a crash with a large vehicle like a bus. The fact that the older participants rated bicycles and electric bicycles as less risky may reflect their ability to deduce the mass, maneuverability and yielding ability of smaller vehicles, recognizing that they could avert crashes more easily than large vehicles and that they might cause less severe injuries.^32-33^ From a prevention perspective, smaller vehicles do in fact present somewhat lesser risk of pedestrian crash than larger vehicles, but they are still risky, and still collide with pedestrians. Prevention programs designed to help pedestrians recognize the risk of pedestrian crash with all moving vehicles, including smaller ones, might prove valuable.

As hypothesized, perception of pedestrian rather than driver responsibility to avoid collisions tended to increase with age. Although the data patterns were not entirely consistent, GEE models offered a pattern of results suggesting young adults and older children attributed greater responsibility to avoid a crash to the pedestrian rather than the driver. This result is sensible: young adults and older children have greater capacity to avoid crashes while walking on the street and therefore might judge crash avoidance as their responsibility to a greater degree than younger children. They may also have a better sense of the ability of drivers to avoid crashes, having driving experience themselves and/or elevated cognitive understanding of the capacity of vehicles to stop and swerve in attempts to avert crashes. Age-graded increases in responsibility may also arise through language and socio-cognitive development, including greater consideration of situational factors^34^ and more developed cognitive judgment, decision-making, impulse control, and understanding of horizontal projectile motion.^30,35-36^ Prevention efforts in this domain might best focus on educating drivers to take particular care around children, and altering the built environment to slow traffic speeds where children frequently cross streets (e.g., near schools).

Our final hypothesis was that participants might judge greater pedestrian responsibility to avert crashes when many vehicles were approaching on the road rather than just a single vehicle. This hypothesis proved true for all vehicle types among fifth/sixth graders, and for all vehicle types except bicycles for university students. The higher risk with multiple vehicles versus a single vehicle approaching reported by fifth/sixth graders but not younger or older children is a bit perplexing, but may possibly result from their rapidly-emerging cognitive ability to distinguish and consider the risk consequences of many approaching vehicles instead of a single approaching vehicle. In fact, perceptions may be exaggerated in this young adolescent age group as the relevant cognitive skills to judge traffic are emerging and they struggle through the adolescent patterns of calibrating risk-taking behavior, both in traffic and more generally in life decisions.^20,37^ The perceptions among university students may reflect their accurate comprehension of the risks involved and the maneuverability capacities of the oncoming vehicles.

Broadly, our findings can be interpreted from the perspective of child and adolescent development. The younger children in our study tended to perceive greater risk as pedestrians in traffic, but to perceive less personal responsibility to reduce that risk than older children and young adults. From a cognitive development perspective, this is logical: young children have reduced ability to keep themselves safe in traffic and therefore perceive greater risk but lesser responsibility. The result may also be interpreted from a social development perspective. Younger children may be socialized by parents to avoid traffic as a risky and dangerous environment, thus increasing their perception of risk but simultaneously creating a feeling that they have less responsibility to be safe in traffic. Instead, that responsibility is socialized to be attributed to drivers and adult pedestrian companions.

Among the eighth grade group, who are amidst brain development changes that might lead to increased risk-taking and decreased perception of crash responsibility,^38-39^ our results were somewhat contrary to the existing literature. We did not find consistent patterns of diminished personal responsibility or increased intentions to take risks among the eighth graders we studied. This may reflect the fact that traffic situations present grave risk of personal harm and therefore are more tangibly dangerous than domains like substance use and sexual risk-taking that are commonly studied in the literature. Additionally, the scenarios presented did not involve peers, whose presence is known to increase risk taking in this age group.^40^ Alternatively, our finding may reflect differences in Chinese versus Western cultures, where the bulk of this research has been conducted.^41-42^

Strengths, Limitations, and Implications. Our study had several strengths. We considered age-related factors that influence perception of risk in pedestrian settings, a factor that is poorly understood and has implications for pedestrian safety worldwide. We conducted our research in China, the country with the highest number of pedestrian injuries and deaths worldwide,^2^ and considered multiple factors – the type and size of vehicle, the number of approaching vehicles, and the age/development and gender of the pedestrian – to identify factors and interactions understudied in the existing literature that may influence how child and adult pedestrians judge the safety of crossing streets, and therefore how we develop prevention programs to reduce pedestrian injury burden.

The study also had limitations. The cross-sectional design prohibits causal inferences, and future research might employ a longitudinal approach to study developmental effects among the same sample of children as they grow. Another weakness was the reliance only on self-report data, which may have triggered demand characteristics and yielded biased results. It also introduces potential biases from common method variance, as we collected only self-reported outcome measures. We collected data in just one Chinese city, and selected research sites (schools and university) using convenient rather than random sampling. Results may vary in other cities, cultures, or countries. Similarly, we limited our analyses to a restricted set of information. We did not consider, for example, potential three-way interactions between one vs. many oncoming vehicle, type of vehicle, and vehicle speed in participants’ judgement of driver vs. pedestrian responsibility to avert crashes. Future research could extend our results in various directions. Finally, we relied on an adult sample of university students, who were still somewhat youthful themselves. Future research might collect data from middle-aged and older adults to evaluate adult developmental factors in the questions of interest.

Our findings have multiple implications for the development of pedestrian safety interventions. Specifically, the results suggest researchers and policy-makers should consider age differences in risk perception and responsibility attribution when designing and implementing interventions to reduce pedestrian-vehicle crashes. Among the younger children we studied, research on specific cognitive/perceptual factors that influence judgment of risk across different vehicle types and traffic situations may lead to targeted training and awareness for children, or to improved adult supervision while crossing roads until age-graded cognitive development occurs. Continued global efforts to protect child pedestrians through reduced speeds in school zones, laws concerning school bus loading and unloading, and pedestrian infrastructure improvements to keep children away from traffic through barriers, overpasses and underpasses, and other such strategies are warranted. Another logical extension of our findings would be to examine factors that might increase perception of pedestrian responsibility to avoid collisions among particular age groups in the future, as greater perceived responsibility is likely to lead to increased self-efficacy to instigate behavior change that improves personal safety.^43-44^

## Conclusion

Study results suggest participants of all ages perceive that bicycles and electric bicycles pose less risk in pedestrian settings than cars and buses, although children perceived greater risks from all vehicles than young adults. We also found that all participants attribute more responsibility to avoid pedestrian-vehicle crashes to pedestrians, although children ascribed less responsibility to pedestrians than did young adults. These findings are valuable to guide design and implementation of age-specific interventions to reduce pedestrian-vehicle crashes. With continued research on the topic, scholars can develop a better understanding of pedestrians’ perception of risk and consider ways to alter those perceptions, ultimately contributing to efforts to mitigate the frequency and severity of unintentional traffic injuries in China and worldwide.
